# Lassa and Crimean-Congo Hemorrhagic Fever Viruses, Mali

**DOI:** 10.3201/eid2505.181047

**Published:** 2019-05

**Authors:** Jan Baumann, Mandy Knüpfer, Judicael Ouedraogo, Brehima Y. Traoré, Asli Heitzer, Bourama Kané, Belco Maiga, Mariam Sylla, Bouréma Kouriba, Roman Wölfel

**Affiliations:** Bundeswehr Institute of Microbiology, Munich, Germany (J. Baumann, M. Knüpfer, A. Heitzer, R. Wölfel);; Centre d’Infectiologie Charles Mérieux du Mali, Bamako, Mali (J. Ouedraogo, B.Y. Traoré, B. Kouriba);; Hôpital du Mali, Bamako (B. Kané);; Centre Hospitalier Universitaire Hospital Gabriel Touré, Bamako (B. Maiga, M. Sylla)

**Keywords:** Lassa virus, LASV, Crimean-Congo hemorrhagic fever virus, CCHFV, viruses, Mali, Bamako, hemorrhagic fever

## Abstract

We report detection of Lassa virus and Crimean-Congo hemorrhagic fever virus infections in the area of Bamako, the capital of Mali. Our investigation found 2 cases of infection with each of these viruses. These results show the potential for both of these viruses to be endemic to Mali.

Numerous viral hemorrhagic fevers are endemic to countries in Africa. Despite the underlying pathogens originating from diverse virus families, clinical features of hemorrhagic fevers are similar, including fever, malaise, abdominal pain, vomiting, headache, and myalgia (*1*). These nonspecific symptoms and their similarity to other infectious diseases common in West Africa, such as malaria, complicate the differential diagnosis. In regions where surveillance data are limited and healthcare workers are less aware of viral hemorrhagic diseases, the possibility for misdiagnosis is high.

Lassa virus (LASV; species *Lassa mammarenavirus*, genus *Arenaviridae*) is the causative agent of Lassa fever. The multimammate rat, *Mastomys* spp., is the natural host of LASV and sheds the virus in urine and droppings. Transmission of LASV to humans usually occurs through contact with the excreta of infected rodents or with body fluids from persons with symptomatic illness.

Lassa fever is known to be endemic in parts of West Africa; most cases are reported from Guinea, Liberia, Sierra Leone, and Nigeria. LASV species in these geographic regions are related genetically, suggesting an ongoing exchange between LASV-endemic regions (*2*). People living in rural areas of West Africa are most at risk for Lassa fever. In recent years, an increasing trend in the number of Lassa fever cases has been observed in countries of West Africa. Annually, ≈300,000 persons are infected with LASV in virus-endemic areas, and ≈5,000 die. Because LASV infections can be asymptomatic, case numbers likely are underestimated (*3*). Although some reports describe serologic evidence that LASV is endemic to Mali (*4**,**5*), few surveillance studies have been conducted. One such study identified a distinct LASV clade in rodents 280 km south of Bamako, the capital of Mali (*6*). In addition, a young man traveling in the border region of Mali and Burkina Faso died from an acute LASV infection in 2009 after returning to the United Kingdom (*7*).

Another prominent pathogen causing hemorrhagic fever, Crimean-Congo hemorrhagic fever virus (CCHFV; family *Nairoviridae*, genus *Orthonairovirus*) is endemic to many regions, including Eurasia, Central America, and parts of Europe and Africa. To date, reports of acute CCHFV infections in West Africa are limited to Senegal (*8*) and Mauritania (*9*). Nevertheless, recent studies confirmed the incidence of CCHFV in *Hyalomma* spp. ticks collected from domestic cattle in southern Mali (*10*). In addition, serologic studies show evidence for human contact with CCHFV (*11*). However, no human cases of acute infection with CCHFV have been identified in Mali. We describe 2 cases of LASV infection and 2 cases of CCHFV infection detected in hospitalized pediatric patients in Bamako.

## The Study

During April 2016–May 2017, we screened malaria-negative blood samples collected at the pediatric department of the University Hospital Gabriel Touré and the pediatric ward of Hôpital du Mali, both located in Bamako. We included febrile patients 3 months to 14 years of age in the study. We obtained ethics approval from the Research Ethics Institutional Review Board, University of Bamako, Mali (no. 2016/01/CE/FMPOS). 

We screened 489 samples for various bacterial and viral pathogens, including LASV and CCHFV. We extracted total nucleic acid using the QIAamp viral RNA kit (QIAGEN, https://www.qiagen.com) and amplified viral RNA by using LASV reverse transcription PCR (*12*) and CCHFV reverse transcription quantitative PCR (*13*) protocols.

We detected LASV RNA in blood samples from 2 patients, a 5-year-old boy and a 13-year-old girl. Both children were treated for episodes of high fever during September–October 2016 at the Hôpital du Mali. The Centre d’Infectiologie Charles Mérieux du Mali in Bamako performed conventional reverse transcription PCR and gel electrophoresis on the LASV. Isolated DNA fragments were shipped to the Bundeswehr Institute of Microbiology (Munich, Germany) for further investigation.

**Figure 1:**
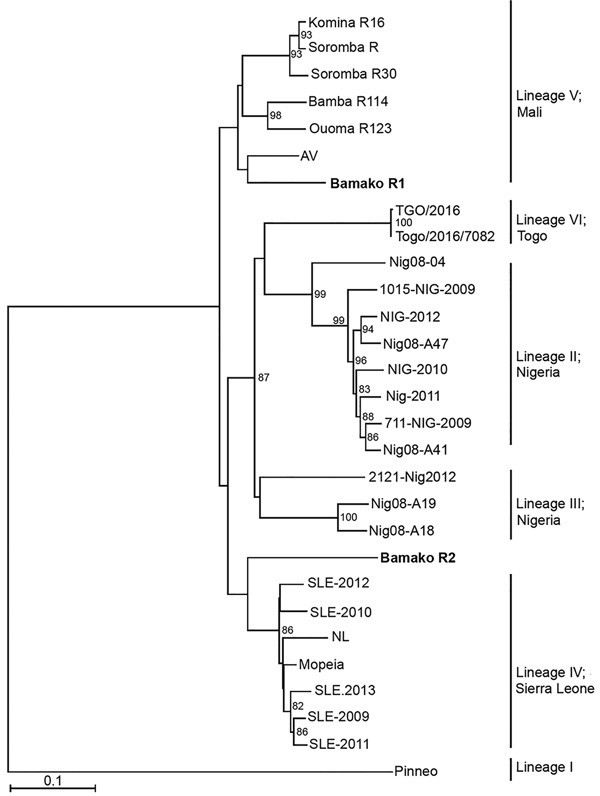
Phylogenetic analysis of representative Lassa virus (LASV) isolates identified in Mali in 2016 (bold) and reference isolates. The tree was constructed by using full-length sequences of the small RNA segment and the neighbor-joining method with bootstrapping to 10,000 iterations. Partial sequences were compared by using the pairwise deletion method. The tree is drawn to scale. Evolutionary analyses were conducted in MEGA7 (https://www.megasoftware.net). Scale bar indicates nucleotide substitutions per site.

Diagnostic amplicons of the LASV small (S) RNA segment, 278 nt from the boy and 276 nt from the girl, were sequenced by Sanger sequencing (GenBank accession nos. MH473586–7). Sequence analysis of the viral S segment showed that 1 patient was infected by a LASV strain that clusters genetically with viruses belonging to lineage IV (Figure 1). Because lineage IV strains previously were not reported to circulate in wildlife in Mali, we suspect this virus reached the country through regional migratory activity of wildlife. Nevertheless, epidemiologic data about LASV in Mali are limited, and frequent exchange between the LASV lineages in West Africa is possible.

The second identified Lassa fever case was caused by a LASV strain belonging to the Mano River clade with 91% similarity to Lassa Soromba R (GenBank accession no. KF478765) (Figure 1). This virus was first isolated in 2010 from rodents near the village Soromba at the southern tip of Mali (*6*). Additional laboratory characterization at that time revealed a relatively mild pathogenicity in macaques (*14*). However, Lassa Soromba R is closely related to the AV strain (GenBank accession no. FR832711) that caused a fatal infection in a young man who returned to Germany after traveling to Ghana, Côte d’Ivoire, and Burkina Faso in 2000 (*15*) and is related to a strain from a patient in the United Kingdom who likely was infected in the border region between Mali and Burkina Faso in 2009 (*7*). Because the case we identified originated in a district of Bamako, we believe LASV could be more widely distributed in southern Mali than previously believed.

**Figure 2:**
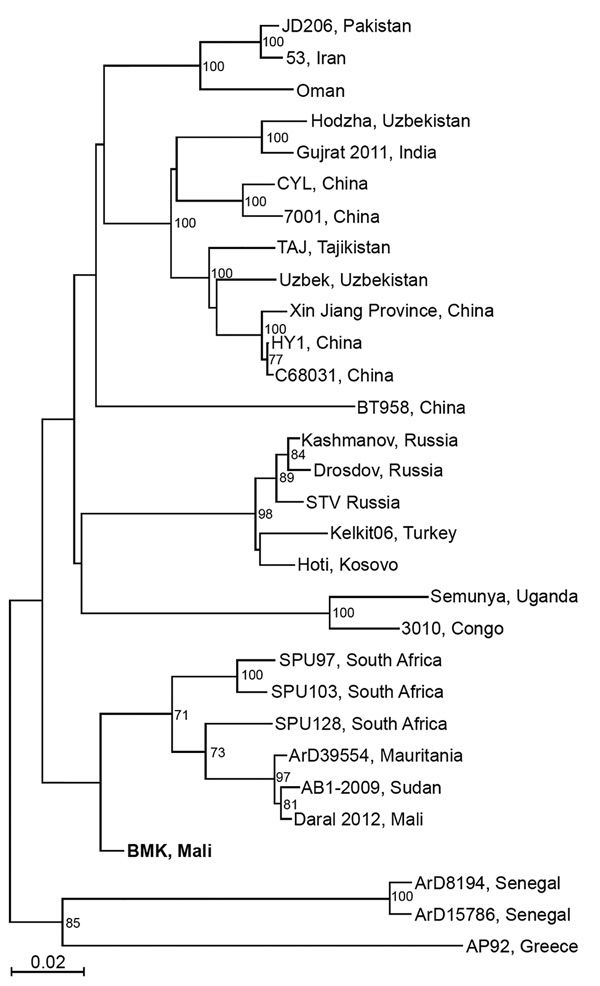
Phylogenetic analysis of representative Crimean-Congo hemorrhagic fever virus (CCHFV) isolates identified in Mali in 2017 (bold) and reference isolates. The tree was constructed by using full-length sequences of the small RNA segment and the neighbor-joining method with bootstrapping to 10,000 iterations. Partial sequences were compared by using the pairwise deletion method. The tree is drawn to scale. Evolutionary analyses were constructed in MEGA7 (https://www.megasoftware.net). Scale bar indicates nucleotide substitutions per site.

We also detected CCHFV, which previously was not known to circulate in the population of Mali. Using reverse transcription quantitative PCR, we detected acute CCHFV infection in 2 patients hospitalized at Hospital Gabriel Touré in April 2017, a 1-year-old boy (cycle threshold 32.74) and a 2-year-old boy (cycle threshold 36.95). We obtained sequence data from the viral S segment for the 1-year-old boy. Phylogenetic analysis showed that this virus is related to CCHFV strain ArD39554 from Mauritania and CCHFV sequence (GenBank accession no. KF793333) recently detected in ticks collected only 25 km from Bamako (*10*) (Figure 2). We were unable to extract sufficient genomic material to perform sequencing on samples from the second case-patient.

## Conclusions

In summary, our study detected 2 cases of infection with LASV in Bamako, Mali, indicating a broader distribution of LASV in Mali than previously known. This finding raises serious public health concerns for future LASV infection in cities in Mali. We also identified 2 human cases of infection with CCHFV in Mali, suggesting an extended CCHFV-endemic region in Africa. Our results underline the need for LASV and CCHFV surveillance programs in sub-Saharan regions of Mali, Burkina Faso, and Niger, which have similar ecology.
